# 15-Deoxy-Δ^12,14^-Prostaglandin J_2_ Inhibits Homing of Bone Marrow-Derived Mesenchymal Stem Cells Triggered by Chronic Liver Injury via Redox Pathway

**DOI:** 10.1155/2015/876160

**Published:** 2015-09-20

**Authors:** Xin Liu, Shuangshuang Jia, Weiyang Li, Le Yang, Lin Yang, Lin Wang, Liying Li

**Affiliations:** Department of Cell Biology, Municipal Laboratory for Liver Protection and Regulation of Regeneration, Capital Medical University, Beijing 100069, China

## Abstract

It has been reported that bone marrow-derived mesenchymal stem cells (BMSCs) have capacity to migrate to the damaged liver and contribute to fibrogenesis in chronic liver diseases. 15-Deoxy-Δ^12,14^-prostaglandin J_2_ (15d-PGJ_2_), an endogenous ligand for peroxisome proliferator-activated receptor gamma (PPAR*γ*), is considered a new inhibitor of cell migration. However, the actions of 15d-PGJ_2_ on BMSC migration remain unknown. In this study, we investigated the effects of 15d-PGJ_2_ on the migration of BMSCs using a mouse model of chronic liver fibrosis and primary mouse BMSCs. Our results demonstrated that *in vivo*, 15d-PGJ_2_ administration inhibited the homing of BMSCs to injured liver by flow cytometric analysis and, *in vitro*, 15d-PGJ_2_ suppressed primary BMSC migration in a dose-dependent manner determined by Boyden chamber assay. Furthermore, the repressive effect of 15d-PGJ_2_ was blocked by reactive oxygen species (ROS) inhibitor, but not PPAR*γ* antagonist, and action of 15d-PGJ_2_ was not reproduced by PPAR*γ* synthetic ligands. In addition, 15d-PGJ_2_ triggered a significant ROS production and cytoskeletal remodeling in BMSCs. In conclusion, our results suggest that 15d-PGJ_2_ plays a crucial role in homing of BMSCs to the injured liver dependent on ROS production, independently of PPAR*γ*, which may represent a new strategy in the treatment of liver fibrosis.

## 1. Introduction

Bone marrow-derived mesenchymal stem cells (BMSCs) are multipotent nonhaematopoietic cells with the ability to differentiate toward a variety of cell types [1, 2]. They have received a great deal of attention as the therapeutic potential for wound healing process [3, 4]. However, it is noticeable that BMSCs have potential to differentiate toward myofibroblasts to exaggerate organ damage. It has been reported that after liver injury, BMSCs could migrate to the damaged liver and become the major origin of hepatic myofibroblasts to promote liver fibrosis by generating extracellular matrix components [5]. Although activated hepatic stellate cells (HSC), residential fibroblasts, circulating fibrocytes, and epithelial-mesenchymal transition (EMT) are proportions of hepatic myofibroblasts, BMSC-derived hepatic myofibroblasts are the overwhelming majority [6]. Considering the importance of BMSCs in liver fibrosis, identification of the molecular mechanism underlying BMSC migration may represent an effective strategy for the treatment of fibrotic liver disease.

BMSC migration can be regulated by a variety of molecules, such as platelet derived growth factor (PDGF), stromal-derived factor-1 (SDF-1), vascular endothelial growth factor (VEGF), and basic fibroblast growth factor (bFGF) [7–10]. In addition, fibroblast activation protein (FAP), Sry-related high-mobility group box 11 (Sox11), and activin B and vitamin C transporter were also involved in the migration of BMSCs [11–14]. Furthermore, our previous results indicated that sphingosine 1-phosphate (S1P) gradient between liver and bone marrow induced migration of BMSCs to the damaged liver [15]. Although much work has already been done to elucidate the mechanistic basis underlying the migration of BMSCs, the potential effects of other endogenous molecules on BMSC migration is still undefined.

15-Deoxy-Δ^12,14^-prostaglandin J_2_ (15d-PGJ_2_), an endogenous ligand for peroxisome proliferator-activated receptor gamma (PPAR*γ*), has been considered a pleiotropic regulator in cell apoptosis, proliferation, and inflammation [16]. In addition, several reports have demonstrated that 15d-PGJ_2_ could inhibit cell migration* in vivo* and* in vitro*. In mouse model of chronic eosinophilia, 15d-PGJ_2_ suppressed eosinophil migration into the peritoneal cavity [17]. 15d-PGJ_2_ also decreased neutrophil migration to the inflammatory site in experimental acute peritonitis [18].* In vitro*, airway smooth muscle cell and mammary cancer cell migration are reduced by 15d-PGJ_2_ treatment [19, 20]. More recently, the function of 15d-PGJ_2_ in the liver fibrosis has garnered much interest, and our previous report has confirmed that 15d-PGJ_2_ administration reduced bone marrow-derived monocyte/macrophage (BMM) migration to the damaged liver and ameliorated liver fibrosis in mouse models [21]. Even though BMSCs are another cell type derived from bone marrow and closely related to liver fibrogenesis, little is known about the effect and the underlying mechanism of 15d-PGJ_2_ on the migration of BMSCs.

The cytoskeleton is a system of intracellular filaments which is crucial for cell shape, division, and other functions in the cell [22]. After tissue injury, a large component of cellular responses is related to the cytoskeleton [23]. For instance, it has been demonstrated that cytoskeletal remodeling plays an important role in cell migration in response to stimulation, including BMSCs [24, 25]. There are reports indicating that 15d-PGJ_2_ could alter cytoskeletal structure of several cell types in which it reduces cell migration [18, 20]. Nevertheless, it is still not clear whether the potential function of 15d-PGJ_2_ in the BMSC migration is linked to the cytoskeleton regulation.

The present study aims to investigate the effect of 15d-PGJ_2_ on BMSC migration triggered by chronic liver injury. Here, we found that,* in vivo*, 15d-PGJ_2_ administration reduced the homing of BMSCs to the injured liver and,* in vitro*, 15d-PGJ_2_ inhibited primary mouse BMSC migration via production of ROS, independently of PPAR*γ*. In addition, the effect of 15d-PGJ_2_ in BMSCs was associated with cytoskeletal remodeling. These results suggest that 15d-PGJ_2_ holds great promise in the treatment of liver fibrosis.

## 2. Materials and Methods

### 2.1. Materials

*α*-MEM was from Invitrogen (Grand Island, NY). Fetal bovine serum (FBS) was from Hyclone/Thermo Scientific (Victoria, Australia). Anti-CD105 and anti-CD166 antibodies used for flow cytometry analysis were from eBioscience (San Diego, CA). Anti-PPAR*γ* antibody used for immunofluorescence was from Santa Cruz Biotechnology (CA, USA). PCR reagents were from Applied Biosystems (Foster City, CA). 15d-PGJ_2_ was from Cayman Chemical (Ann Arbor, MI). 2′,7′-Dichlorohydrofluorescein diacetate (DCFH-DA) was from Molecular Probes (Interchim, France). Troglitazone and ciglitazone were from Biomol (Tebu, France). GW9662, N-acetylcysteine (NAC), and other common reagents were from Sigma (St. Louis, MO).

### 2.2. BMSCs Preparation

Bone marrow (BM) cells were isolated from BM of ICR mice (closed colony mice) aged 3 weeks by flushing the tibias and femurs (Laboratory Animal Center, Capital Medical University) with a 25-gauge needle. Then, the cells were passed through 70 mm nylon mesh and washed with PBS containing 2% FBS for three times. BMSCs were cultured as described previously [5]. In brief, BM cells were cultured with *α*-MEM containing 20% FBS at 37°C in 5% CO_2_ for 1 week. The culture medium was replaced twice a week to remove the nonadherent cells. After the first subculture, *α*-MEM containing 15% FBS was used to culture BMSCs. BMSCs were characterized by flow cytometry analysis, and passage 3 to passage 5 were used in the experiments. All animal work was performed under the ethical guidelines of the Ethics Committee of Capital Medical University.

### 2.3. Mouse Models

ICR mice aged 6 weeks received intraperitoneal injections of 1 *μ*L/g body weight of CCl_4_/olive oil (OO) mixture, 1 : 9 v/v, or OO singly, twice per week. 15d-PGJ_2_ (0.3 mg/kg body weight) or saline firstly was administrated the day before CCl_4_ or OO treatment and then twice per week before CCl_4_ or OO treatment for 4 weeks (*n* = 7 per group).

Another group of ICR mice received lethal irradiation (8 Grays) and then immediately received transplantation by a tail-vein injection of 1.5 × 10^7^ whole BM cells obtained from 3-week-old enhanced green fluorescent protein (EGFP) transgenic mice. 4 weeks later, mice received intraperitoneal injections of CCl_4_ or OO twice per week for 4 weeks. 15d-PGJ_2_ (0.3 mg/kg body weight) or saline firstly was administered the day before CCl_4_ or OO treatment and then twice per week before CCl_4_ or OO treatment for 4 weeks (*n* = 7 per group).

### 2.4. Immunofluorescence and High Content Analysis

Cultured BMSCs with or without treatments were fixed in 4% paraformaldehyde in PBS for 30 minutes. Then cells were washed twice with PBS, permeabilized in 0.5% TritonX-100 in PBS for 15 minutes, blocked with 2% BSA for 1 hour, and then incubated with anti-PPAR*γ* antibody (1 : 100), followed by incubation of secondary antibody conjugated with Cy3 (1 : 100; Jackson ImmunoResearch Laboratories, West Grove, PA). Filamentous actin (F-actin) was stained with FITC-conjugated phalloidin (1 : 80, Molecular Probes, Eugene, OR) for 20 minutes. The nuclei were stained with DAPI and 50 *μ*L PBS was left in each well. The plates were imaged on a Thermo Scientific CellInsight personal cell imaging (PCI) platform (Cellomics, Inc., Thermo Fisher Scientific Inc., Waltham, MA), with a ×10 objective using the Thermo Scientific Cellomics iDEV software. Thirty-six fields were automatically acquired by the software, corresponding to at least 3,000 cells. The total Cy3 or FITC fluorescence intensity of each well was analyzed by Cellomics Cell Health Profiling BioApplication software.

### 2.5. Fluorescent Measurement of Intracellular Reactive Oxygen Species

BMSCs were plated in the wells of 96-well plates (Corning, NY) and allowed to attach overnight in *α*-MEM. Cells were then loaded for 15 minutes at 37°C with 5 *μ*M DCFH-DA in *α*-MEM without FBS. After two washings with PBS, BMSCs were treated with 15d-PGJ_2_ (1, 2, or 5 *μ*M) or vehicle, and the fluorescence intensity of each well was determined after 5, 10, 15, 20, and 30 minutes by high content analysis.

### 2.6. Flow Cytometric Analysis

Nonparenchymal cells (NPCs) of mouse liver were isolated as described by Han et al. [21]. Cultured BMSCs were prepared to achieve single cell suspensions. The cells were resuspended at 1.5 × 10^6^ cells/100 *μ*L in PBS and then incubated with PE-CD105 (1 : 40), PE-CD166 (1 : 80), or their isotype-matched negative control antibodies. After incubation in the dark for 15 minutes, the cells were washed with PBS and subjected to flow cytometric analysis. Flow cytometric analysis was performed on a FACS Aria and analyzed with FACS Diva4.1 (BD Biosciences).

### 2.7. Cell Migration Assay

BMSC migration was determined by Boyden chambers as described previously by Liu et al. [26]. Briefly, BMSCs were serum starved for 24 hours and then exposed to 15d-PGJ_2_, troglitazone, ciglitazone, or vehicle for 12 hours. Then 4 × 10^4^ BMSCs were seeded to the upper chamber. Cell migration was allowed to proceed for 4 hours at 37°C in 5% CO_2_. BMSCs migrating to the lower face of the porous membrane were fixed with cold methanol for 30 minutes and stained with hematoxylin for 1 hour. BMSCs on the upper membrane surface were removed with cotton swabs. Migrated BMSCs were photographed in at least six random fields per filter and quantified by cell counting.

### 2.8. Real-Time RT-PCR

Total RNA was extracted from frozen liver specimens or cultured BMSCs with or without treatments, using an RNeasy kit (Qiagen, Hilden, Germany). Real-time RT-PCR was performed with an ABI Prism 7300 sequence-detecting system (Life Technologies, Foster City, CA), as described previously [15]. Primers (MWG Biotech, Ebersberg, Germany) used for real-time RT-PCR were as follows: 18 s rRNA, sense, 5′-GTA ACC CGT TGA ACC CCA TT-3′, and antisense, 5′-CCA TCC AAT CGG TAG TAG CG-3′; PPAR*γ*: sense, 5′-GCC CAC CAA CTT CGG AAT C-3′, and antisense, 5′-TGC GAG TGG TCT TCC ATC AC-3′.

### 2.9. Statistical Analysis

All results were confirmed at least by three independent experiments. The results are expressed as mean ± SEM. Statistical significance was determined by Student's *t*-test or ANOVA. Statistical significance was defined as *P* < 0.05.

## 3. Results

### 3.1. 15d-PGJ_2_ Inhibits Homing of BMSCs to the Injured Liver

**Figure 1:**
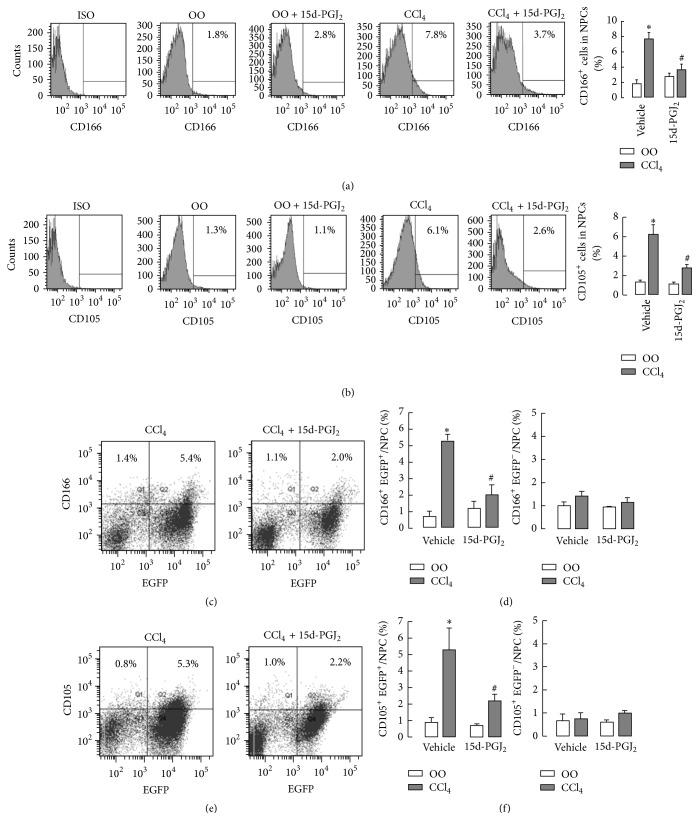
15d-PGJ_2_ inhibits the migration of BMSCs toward injured liver. ((a) and (b)) 4 weeks of CCl_4_ were used to induce mouse liver fibrosis with or without 15d-PGJ_2_ administration (*n* = 7 per group). Total MSCs were isolated from the NPCs in the liver by flow cytometry analysis based on CD166 and CD105. Representative histograms and proportions of CD166^+^ (a) and CD105^+^ (b) in NPCs. ((c)–(f)) Mice were lethally irradiated and received whole BM transplants from EGFP transgenic mice, followed by CCl_4_ injection for 4 weeks in the presence or absence of 15d-PGJ_2_ treatment. BMSCs in the liver NPCs were identified as CD166^+^/EGFP^+^ or CD105^+^/EGFP^+^. ((c) and (e)) Representative histograms of BMSCs. ((d) and (f)) The proportions of CD166^+^/EGFP^+^ BMSCs and CD166^+^/EGFP^−^ resident MSCs (d) and CD105^+^/EGFP^+^ BMSCs and CD105^+^/EGFP^−^ resident MSCs in NPCs of liver tissues. ^*∗*^ *P* < 0.05, compared with olive oil (OO) group. ^#^ *P* < 0.05, compared with CCl_4_ group without 15d-PGJ_2_ (*n* = 7).

We previously have confirmed that 15d-PGJ_2_ could inhibit homing of BMM to the damaged liver tissue in mouse model of chronic liver injury [21]. Although BMSCs are also known to migrate to the injured liver in this process, whether it could be regulated by 15d-PGJ_2_ has not been elucidated. To investigate the effect of 15d-PGJ_2_, we first used CCl_4_ injection to induce mouse liver fibrosis. Four weeks later, NPCs in liver tissues were analyzed by flow cytometric analysis, and total MSCs were characterized as positive for markers CD166^+^ or CD105^+^. The results showed that 15d-PGJ_2_ administration significantly decreased the proportion of total MSCs (CD166^+^ or CD105^+^ cells) in liver NPCs compared with that in the liver without 15d-PGJ_2_ treatment (Figures 1(a) and 1(b)).

MSCs are multipotential nonhematopoietic progenitor cells that can be obtained from several tissues, including the bone marrow (BMSCs) and the liver tissue (L-MSCs). We next want to examine whether these decreased MSCs by 15d-PGJ_2_ are bone marrow derived or resident MSCs. For this purpose, we reconstituted BM in the irradiated mice by transplantation of the genetic EGFP-labeled BM cells. Liver fibrosis was also induced by CCl_4_ administration for 4 weeks with or without 15d-PGJ_2_ treatment. BMSCs in the liver were isolated and counted as double positive for CD166/EGFP and CD105/EGFP, respectively. The results indicated that, in liver NPCs, there was no significant difference in the proportions of resident MSCs (CD166^+^/EGFP^−^ or CD105^+^/EGFP^−^) in the 15d-PGJ_2_-treated mice compared with 15d-PGJ_2_ nontreatment group (Figures 1(c)–1(f)). However, the proportions of CD166^+^/EGFP^+^ and CD105^+^/EGFP^+^ BMSCs in the damaged liver were markedly decreased by 15d-PGJ_2_ administration (Figures 1(c)–1(f)). These results suggested that 15d-PGJ_2_ inhibited migration of BMSCs to the damaged liver tissue but had no influence on liver resident MSCs in the model of CCl_4_-induced liver injury.

### 3.2. 15d-PGJ_2_ Inhibits Migration of BMSCs* In Vitro*

**Figure 2:**
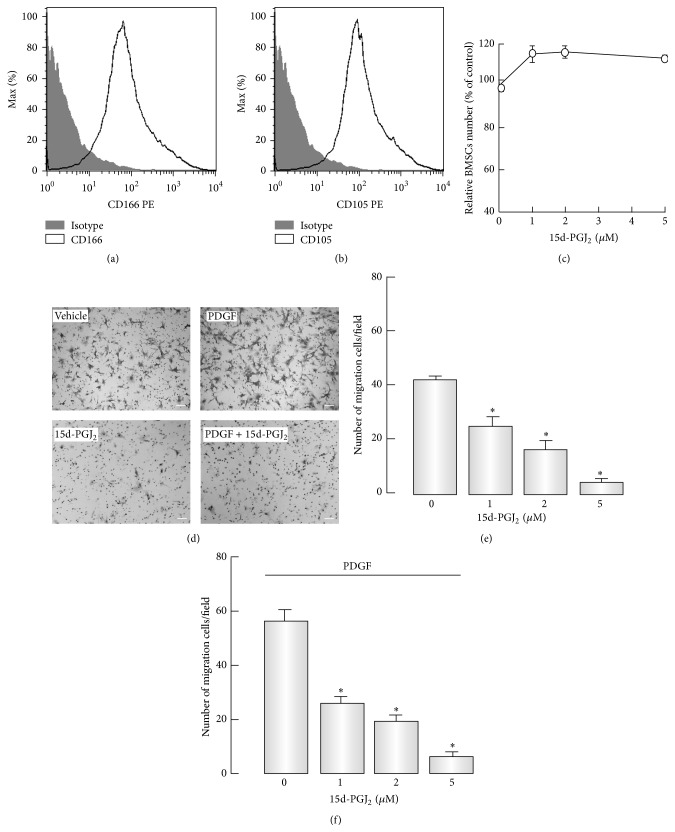
Effects of 15d-PGJ_2_ on BMSCs migration and proliferation. ((a) and (b)) Flow cytometry analysis was performed to identify BMSCs based on the specific marker of CD166 (a) and CD105 (b). (c) BMSCs were exposed to varying concentrations of 15d-PGJ_2_ for 24 hours. Cell Counting Kit-8 was used to assess cell proliferation. ((d)–(f)) Serum-starved BMSCs were preincubated with indicated doses of 15d-PGJ_2_ for 12 hours and then allowed to migrate for 4 hours with or without PDGF (30 ng/mL) administration. Representative images of migration of BMSCs (d). Quantitative analysis of BMSC migration in the presence (f) or absence (e) of PDGF. ^*∗*^ *P* < 0.05, compared with control (*n* = 8). Scale bars, 50 *μ*m.

To further investigate the effect of 15d-PGJ_2_ on BMSC migration* in vitro*, we first performed flow cytometric analysis to identify the purity of BMSCs isolated from mouse BM. The results showed that these cells were predominantly positive for CD166 and CD105 (Figures 2(a) and 2(b)). Herein, these primary BMSCs were performed in the subsequent studies. Transwell migration assay indicated that 15d-PGJ_2_ (1–5 *μ*M) caused a powerful dose-dependent decrease in the migration of BMSCs (Figures 2(d) and 2(e)). In that studies have proved PDGF possess promigration property in liver diseases [27–29], we utilized PDGF to investigate BMSC migration in response to pathological stimuli. The results indicated that PDGF could augment migration of BMSCs, which was also inhibited by 15d-PGJ_2_ in a concentration-dependent manner (Figures 2(d) and 2(f)). Given that 15d-PGJ_2_ possesses proapoptotic and growth inhibitory potential, we determined the effect of 15d-PGJ_2_ on BMSC proliferation using the Cell Counting Kit-8. As shown in Figure 2(c), 15d-PGJ_2_ did not alter the number of living BMSCs at concentrations ranging from 1 to 5 *μ*M. These observations suggested that 15d-PGJ_2_ suppressed BMSC migration not only under normal condition but also in the context of pathological condition.

### 3.3. 15d-PGJ_2_ Inhibits BMSCs Migration via ROS-Dependent Pathway, Independently of PPAR*γ*

**Figure 3:**
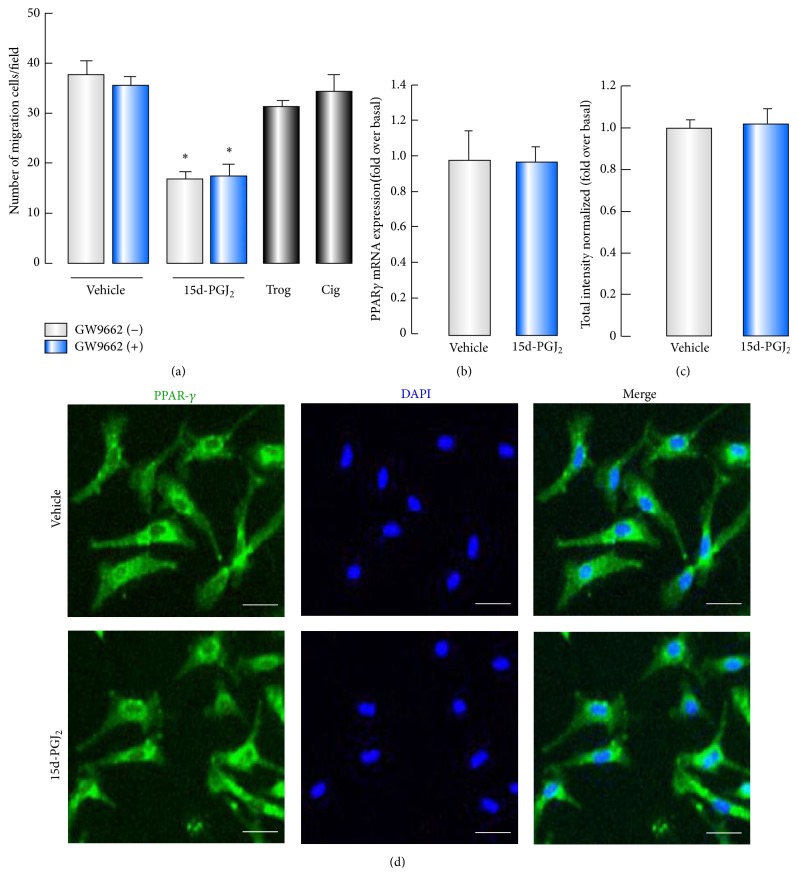
15d-PGJ_2_ inhibits BMSCs migration independently of PPAR*γ*. (a) Serum-starved BMSCs were pretreated with or without 10 *μ*M GW9662 for 1 hour and then exposed to 5 *μ*M 15d-PGJ_2_ or 10 *μ*M troglitazone (Trog) or ciglitazone (Cig) for 12 hours and were subsequently allowed to migrate for 4 hours. ((b)–(d)) Serum-starved BMSCs were incubated with 15d-PGJ_2_ (5 *μ*M) for 12 hours. Real-time RT-PCR was performed to determine mRNA expression of PPAR*γ* in BMSCs (b). PPAR*γ* protein expression was detected by immunofluorescence analysis (green) (d), and total fluorescence intensity of PPAR*γ* was counted with high content analysis (c). ^*∗*^ *P* < 0.05, compared with control (*n* = 6). Scale bars, 50 *μ*m.

It is well revealed that 15d-PGJ_2_ can act through PPAR*γ* pathway [30–32]. Next, we evaluate whether the suppressive effects of 15d-PGJ_2_ on BMSC migration were mediated by PPAR*γ*. The results showed that application of synthetic ligands of PPAR*γ* (troglitazone or ciglitazone) had no effects on BMSC migration (Figure 3(a)). In addition, pretreatment with GW9662 (an irreversible PPAR*γ* antagonist) did not influence the inhibitory effect of 15d-PGJ_2_ (Figure 3(a)). Furthermore, 15d-PGJ_2_ had no influence on PPAR*γ* mRNA in BMSCs (Figure 3(b)). Immunofluorescence was also performed to study the effect of 15d-PGJ_2_ on the protein expression of PPAR*γ*. The results indicated that there was comparable extent of immunoreactivities for PPAR*γ* in the vehicle- or 15d-PGJ_2_-treated BMSCs (Figure 3(d)). High content analysis showed that the fluorescence intensities of PPAR*γ* did not achieve statistical significance among the two groups (Figure 3(c)).

**Figure 4:**
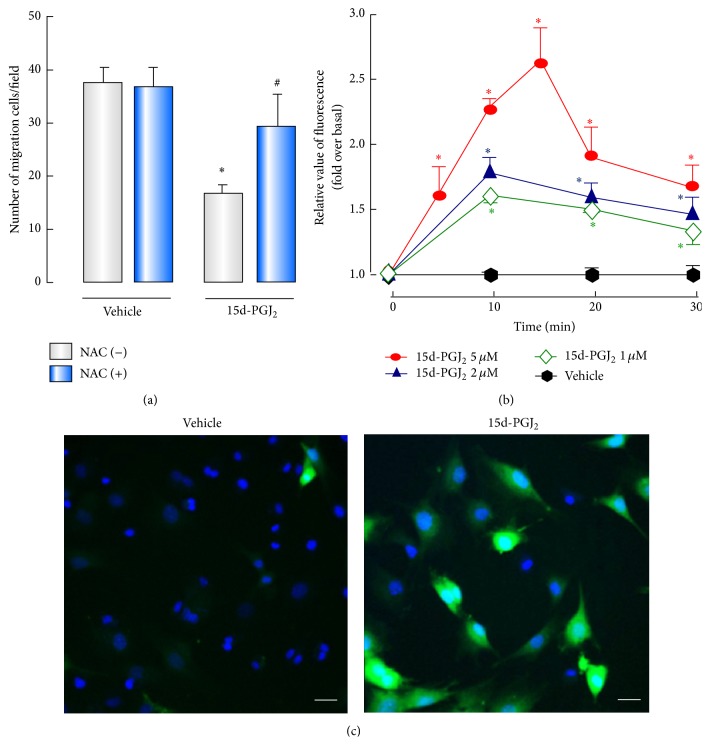
The suppressive effect of 15d-PGJ_2_ on BMSCs migration occurs via ROS production. (a) Serum-starved BMSCs were incubated with 5 *μ*M 15d-PGJ_2_ with or without 2.5 mM NAC (inhibitor of ROS) for 1 hour before treatment and then determined the effect of 15d-PGJ_2_ on BMSCs migration. ((b) and (c)) BMSCs were loaded with DCFH-DA and further treated with 15d-PGJ_2_ at different dose levels and durations. Representative images of fluorescence for ROS (green) in BMSCs (c). Fluorescence was measured using high content analysis (b). ^*∗*^ *P* < 0.05, compared with control. ^#^ *P* < 0.05, compared with 15d-PGJ_2_ group without NAC (*n* = 6). Scale bars, 50 *μ*m.

Recent studies indicate that besides activation of PPAR*γ* ROS production is another mechanism by which 15d-PGJ_2_ elicits its effects [21, 33, 34]. Then we used antioxidant NAC to assess the role of ROS in the inhibitory action caused by 15d-PGJ_2_. We found that preincubation with NAC eliminated the suppressive effect of 15d-PGJ_2_ on BMSC migration (Figure 4(a)). The production of intracellular ROS in response to 15d-PGJ_2_ was further confirmed by employing the peroxide-sensitive probe DCFH-DA prior to the addition of 15d-PGJ_2_. As shown in Figure 4(c), application of 15d-PGJ_2_-induced a significant rapid and transient increase in ROS production, which was in a dose-dependent fashion counted with high content analysis (Figure 4(b)). These results emphasized the importance of ROS in the inhibitory function of 15d-PGJ_2_ on BMSC migration, but not PPAR*γ*.

### 3.4. 15d-PGJ_2_ Inhibiting Migration of BMSCs Requires F-Actin Remodeling

**Figure 5:**
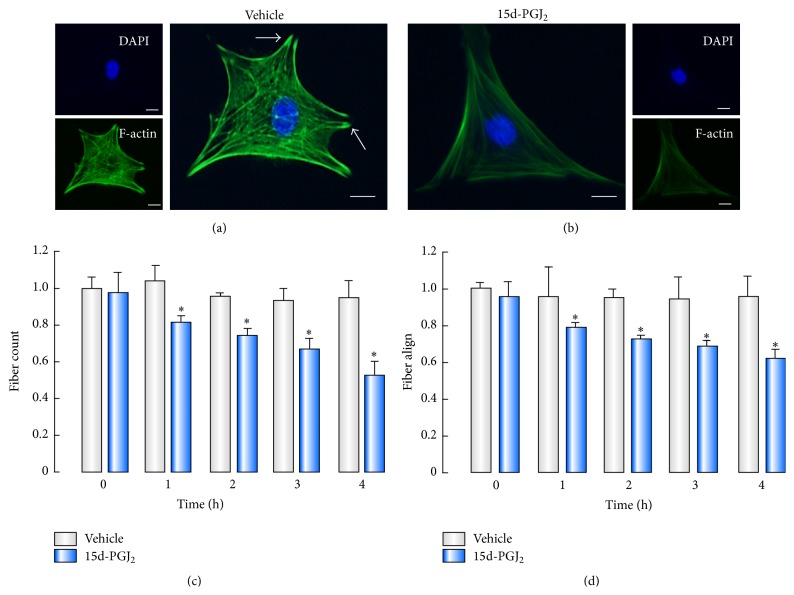
The effect of 15d-PGJ_2_ on F-actin remodeling in BMSCs. ((a) and (b)) BMSCs were incubated with FITC-conjugated phalloidin to identify F-actin (green) in the absence (a) or presence (b) of 15d-PGJ_2_ (5 *μ*M). Focal adhesion-like structures were shown as arrows in (a). ((c) and (d)) High content analysis was used to determine the number of fibers (c) and fiber alignment (d) in BMSCs after 15d-PGJ_2_ treatment at the indicated time points. ^*∗*^ *P* < 0.05, compared with control (*n* = 6). Scale bars, 50 *μ*m.

In response to external stimulation, intracellular signals induce a dynamic remodeling of actin cytoskeleton, which results in changing cell shape and affecting cell motility [35]. It has been demonstrated that the number of actin fibers constitutes a principal profile related to migratory capacity [36, 37]. In addition, actin alignment in cell that represents order extent of fibers alters under different conditions. Herein, we further investigated the change of F-actin with FITC-conjugated phalloidin in BMSCs in the presence or absence of 15d-PGJ_2_. As shown in Figure 5(a), under normal condition, BMSCs showed a migratory phenotype with abundant actin fibers and focal adhesion-like structures could be observed on the edge of cell membrane. Application of 15d-PGJ_2_-induced a static phenotype with fewer actin fibers (Figure 5(b)). In particular, focal adhesion-like structures were disassembled in 15d-PGJ_2_-treated BMSCs (Figure 5(b)). Furthermore, high content analysis was used to determine the amount and distribution of actin fibers in BMSC. The results showed that 15d-PGJ_2_ administration time-dependently decreased number of fibers (Figure 5(c)) and fiber alignment (Figure 5(d)) in BMSCs. These data indicated that 15d-PGJ_2_ could induce F-actin remodeling which were possibly associated with inhibitory action of 15d-PGJ_2_ on BMSC migration.

## 4. Discussion

Earlier reports have documented that 15d-PGJ_2_ exhibits inhibitory effect on migration of several cells; however, it is still under investigation whether 15d-PGJ_2_ plays an important role in migration of BMSCs, the main origin of hepatic myofibroblasts. In the current study, we investigated the effects of 15d-PGJ_2_ on mouse BMSC migration* in vivo* and* in vitro* and the underlying mechanisms. We found that 15d-PGJ_2_ could reduce homing of BMSC triggered by chronic liver injury. In addition, 15d-PGJ_2_ inhibits migration of BMSCs* in vitro*, which is mediated by ROS production, independently of PPAR*γ*. Meanwhile, the suppressive effect of 15d-PGJ_2_ on BMSC migration was associated with cytoskeletal remodeling.

There are two main mechanisms by which 15d-PGJ_2_ carries out its functions. Earlier report has shown that, as an endogenous ligand for PPAR*γ*, 15d-PGJ_2_ could activate PPAR*γ* to regulate target genes expression [38]. In addition, 15d-PGJ_2_ can change cellular redox status, such as production of ROS by forming covalent adducts with cysteine thiols via Michael addition due to its electrophilic *α*, *β*-unsaturated carbonyl group [39]. The effects of oxidative stress on cell migration remain controversial. Although considerable evidence points that ROS promotes migration of several cell types through direct or indirect interactions with migration-related molecules, including focal adhesion kinase (FAK), Rho GTPases, and mitogen activated protein kinase (MAPK) family of signaling pathways, some studies found oxidative stress also could exert suppressive function in cell migration [13, 40–45]. Here, ROS mediated the inhibitory effect of 15d-PGJ_2_ on BMSC migration, eliminated by antioxidant NAC. 15d-PGJ_2_ reduced another bone marrow-derived cell, BMM migration also via formation of ROS in our previous report [21]. Furthermore, in mammary cancer cells, a redox signaling pathway was involved in 15d-PGJ_2_-induced focal adhesion disassembly and F-actin cytoskeletal changes in which 15d-PGJ_2_ attenuated migration [20].

Evidence suggests that PPAR*γ* also participates in cell migration. For instance, 15d-PGJ_2_ suppresses eosinophil migration by activating PPAR*γ* [17]. In addition, PPAR*γ* is involved in 15d-PGJ_2_-induced inhibition of migration in human airway smooth muscle cell and neutrophil [18, 19]. However, in the present study, PPAR*γ* does not mediate the repressive function of 15d-PGJ_2_ in BMSC migration. The effect of 15d-PGJ_2_ was neither reproduced by PPAR*γ* synthetic agonists nor blocked by PPAR*γ* antagonist. In addition, 15d-PGJ_2_ did not affect PPAR*γ* content in the BMSCs. These results suggest that the underlying molecular mechanism of 15d-PGJ_2_ on cell migration is complex and highly cell type specific.

High content analysis is a novel method, which could provide precise statistics according to cells selected for analysis [46]. In addition to detecting PPAR*γ* protein and ROS production in BMSCs on the basis of labeled fluorescence, high content analysis also can measure F-actin remolding in BMSCs. It has been reported that cytoskeletal rearrangement of actin plays a central role in cell migration [24]. In the present study, 15d-PGJ_2_ treatment caused a decrease in the number of fibers and fiber alignment. These findings suggest that 15d-PGJ_2_-mediated actin remodeling is possibly involved in the inhibition of BMSC migration. Similar to our results, cytoskeletal organization is altered in 15d-PGJ_2_ stimulated breast cancer cells (MCF-7) mediated through a mechanism unrelated to PPAR*γ* transcriptional activation [47]. In addition, disruption of F-actin reorganization resulted in the reduction of migration in human MSCs [48]. Furthermore, inhibition of actin polymerization markedly suppressed migration of ovarian cancer cells [49]. In particular, we found 15d-PGJ_2_ treatment disassembled focal adhesion-like structures in BMSCs. Although it is known that 15d-PGJ_2_-induced focal adhesion disassembles via a redox pathway in mammary cancer cells [20], the underlying mechanisms for the regulatory effects of 15d-PGJ_2_ on adhesion-like structures in BMSCs still require further investigation.

## 5. Conclusions

In summary, our results indicate that 15d-PGJ_2_ inhibits homing of BMSCs toward injured liver, and 15d-PGJ_2_ reduces BMSC migration through ROS production and cytoskeletal remodeling, independently of PPAR*γ*. Therefore, we provide a novel regulatory mechanism of BMSC migration and suggest that 15d-PGJ_2_ may be used as an antifibrotic agent during liver fibrosis.
